# A Qualitative Study of How Teens in Washington State Make Sense of Cannabis Edibles Warning Labels and Packaging

**DOI:** 10.1111/dar.70071

**Published:** 2025-11-08

**Authors:** Jessica Fitts Willoughby, Stacey J. T. Hust, Soojung Kang, Leticia Couto, Ron Price, Christina Griselda Nickerson, Opeyemi Johnson, Sarah Ross‐Viles

**Affiliations:** ^1^ The Edward R. Murrow College of Communication, Washington State University Pullman Washington USA; ^2^ College of Communication, DePaul University Chicago Illinois USA; ^3^ Integrated Media Department Gonzaga University Spokane Washington USA; ^4^ Public Health—Seattle and King County Seattle Washington USA; ^5^ School of Public Health, University of Washington Seattle Washington USA

**Keywords:** cannabis, edibles, food packaging, teens, warning labels

## Abstract

**Introduction:**

Washington state's adult use cannabis market operates under regulations by the Washington State Liquor and Cannabis Board to restrict access and promotion among young people. Cannabis edibles sold in the state are required to contain specific labels that inform consumers that the product contains cannabis and provide contact information for Poison Control. However, it is unclear how teens perceive such labels.

**Methods:**

Ten focus groups were conducted with a diverse sample of 28 teens (*M =* 15.93, SD = 1.25) in Washington state, United States. After viewing images of cannabis edible products available in Washington state, participants shared their thoughts and opinions about the packaging, warning labels and nutrition information.

**Results:**

Through a thematic analysis, we noted that teens may be misinterpreting warning labels, and they think warning labels are hidden or unnoticeable. Most teens paid little attention to nutrition labels and often found serving size information confusing. Teens said if an edible product looked similar to snack products they know, they might perceive them as less risky and more enticing. Knowledge of cannabis products also impacted teens' understanding of edible product packaging.

**Discussion and Conclusions:**

Labels alert teens to the fact that products contain cannabis. However, teens often feel such labels apply to younger children and would not keep teens from using a product. Youth might benefit from additional guidance around interpreting cannabis packaging and labels.

**Summary**

- Teens may interpret cannabis edibles warning labels as not relevant to them, but relevant to younger children.
- Teens perceive that packaging characteristics can obscure warning labels on cannabis edibles.
- Teens said they do not often pay attention to nutrition labels and serving sizes.

## 1 Introduction

In 2012, Washington state was one of the first states (with Colorado) to legalise adult‐use cannabis for people ages 21 and older [1]. This means teens have grown up in a state in which the purchase and use of cannabis have always been legal at the state level. Although not federally legal in the United States, as of 2024, 24 states, Washington D.C. and Guam legalised cannabis for recreational use [2]. This has resulted in states determining how to legalise and regulate the sale of cannabis, including making decisions around labelling guidelines and restrictions.

### 1.1 Youth and Cannabis Risks

Research identifies adolescence as a vulnerable period for cannabis use. Adolescent use of cannabis is associated with negative health outcomes. Cannabis use during adolescence is associated with an increased risk of major depression and suicidality as young adults [3]. Brain differences based on cannabis use, including declines in IQ in comparison to non‐cannabis using peers, have been noted, although these relationships are complex and may be impacted by other factors (e.g., environment; [4]). Specific cannabis effects, such as the negative effects of cannabis use on working memory and attention, may be less pronounced when examining an adult sample of participants but are found with younger samples. Age of onset is important to consider, as research has found larger effects for earlier cannabis use [5]. Among people who started using cannabis before age 18, 67% started using it between the ages of 15 and 17, highlighting the importance of prevention efforts geared toward teens [6].

### 1.2 Youth and Cannabis Edibles

Although some teens may choose to use cannabis, some teens and children may also experience accidental use of cannabis, particularly with edibles. Among children ages five and under, accidental exposure to cannabis edibles has increased since the legalisation of cannabis [7, 8]. This phenomenon is disconcerting, given that cannabis edibles can be more easily consumed compared to other forms of cannabis, like the ones that can be inhaled [9]. Furthermore, cannabis edibles may appeal to youth because they are offered in snack‐like forms, such as gummies and candies, and often with enticing packaging designs [10]. Among teens, edible users tend to be heavier cannabis users compared to non‐edible users and started using at a younger age [11].

### 1.3 Cannabis Warning Labels

As more U.S. states legalise cannabis for non‐medical use [12], warning labels and nutrition labels are used to provide consumers with pertinent product information. Cannabis health warnings are an important strategy to share risk information with potential consumers [13]. According to the Elaboration Likelihood Model, people process information through two possible routes, the central route and the peripheral route [14]. Central route processing means people elaborate on the decision and decisions made through this route can lead to more sustained attitude change and persuasion. Peripheral route processing, however, tends to rely on heuristics or cues, such as perceived similarity or product attractiveness [14]. To process messages centrally, people need to be motivated and have the ability to process the message. Health warning labels may be processed by young people through the central route [15], meaning that they consider the arguments presented in the message to inform their attitudes and beliefs.

Past research related to health warning labels in other domains, such as for alcohol and food products, has found that health warning labels can reduce the selection of targeted products in comparison to products with no warning labels [16]. On tobacco products, pictorial warnings in comparison to text‐only warnings have been found to positively impact attention and recall, warning reactions, such as through cognitive elaboration to the warning, attitudes and beliefs, and intentions to not start smoking or quit smoking [17]. In the context of cannabis, experimental research has found benefits to pictorial warnings in comparison to text‐only warnings [18, 19], with Yang and colleagues' work only finding effects for pictorial warnings (as opposed to no warning or a textual warning) to reduce product appeal among adolescents, although both pictorial and textual warnings reduced intentions to use [19].

In a study of Canadian youths, health warning labels were found to be a helpful reminder of the adverse health effects and lowered youths' perceptions that cannabis products were appealing [20]. Also, such warning labels take up space on packaging that may otherwise be used for promotional content that appeals to consumers. Past work looking at appeals in cannabis packaging has found such packaging can increase product appeal among young people, and that plain packaging that included warning labels was viewed as less appealing than packaging that had branding or without health warning labels [21]. However, research indicates that better readability and clarity are needed, including for all audiences [22, 23]. Further, a study with Canadian young adults found that participants wanted packaging that promoted safe consumption among adults and protected children from consumption, such as by providing information on standardised THC units [23].

### 1.4 Washington State Packaging Guidelines

Currently, Washington state provides guidelines that require cannabis products to include two warning labels on cannabis edibles packaging. A symbol called ‘Not For Kids’ consists of a red logo on a white background, showing the drawing of a hand, the ‘Not For Kids’ phrase, and the phone number to Washington Poison Control. Another required label is the Washington Universal Symbol, which consists of a yellow diamond shape with a cannabis leaf on it and the text ‘21 + ’. The Washington Universal Symbol indicates that the product should only be consumed by those who are 21 years old and older [24] and lets consumers know the product contains tetrahydrocannabinol (THC), the primary psychoactive cannabinoid in cannabis [25]. Other U.S. states, such as Colorado and California, require similar warning labels on cannabis edibles packaging [26, 27], although a review of regulations for nonmedical cannabis warning labels required on product packages across 20 states found that requirements varied widely across states [28]. In Washington State, cannabis edibles are also required to include warning statements including: ‘Warning—May be habit forming; Unlawful outside Washington State’; ‘It is illegal to operate a motor vehicle under the influence of cannabis’ and ‘Caution: Intoxicating effects may be delayed by 2+ hours’ [29]. Cannabis edibles may also come in various sizes, with varying dosages and potency. Washington state requires cannabis edibles to include information on the serving size and number of servings in the unit [29]. Additionally, products need to provide information on the total THC included.

Although previous research has provided suggestions for cannabis packaging, the perceptions of U.S. adolescents on the warning labels on cannabis packaging are largely unknown. Given this, we were interested in furthering our understanding of how young people make sense of such messaging. Therefore, the current study endeavoured to answer the following key questions: (i) How do teens make sense of warning labels on cannabis products?; and (ii) How do teens make sense of the serving sizes described on cannabis products?

## 2 Methods

### 2.1 Sample

Teens were recruited through contact with established youth groups in Washington state and snowball sampling to participate in virtual focus groups on Zoom. Twenty‐eight teens from 9 counties participated in this study. The Washington State Institutional Review Board determined the project exempt, and teens provided assent after parents provided consent to participate. We conducted seven focus groups with 3–5 participants each and three in‐depth interviews. We intentionally kept focus groups small to facilitate in‐depth conversations. See Table 1 for demographics. The average age was 15.93 (SD = 1.25). Our sample was diverse as 25% (*n* = 7) identified as non‐heterosexual, and approximately two‐thirds (64.3%, *n* = 18) received free or reduced lunch at school, a proxy for socioeconomic status. Additionally, almost half (46.4%, *n* = 13) of participants identified as non‐White.

**TABLE 1:** Participant demographic characteristics.

Sample characteristics	*n*	%	*M*	SD
Age	28		15.93	1.25
Gender				
Male	9	32.1		
Female	17	60.7		
Non‐binary	1	3.6		
Prefer not to say	1	3.6		
Race				
White	15	53.6		
African American/Black	5	17.9		
Latino/Latina or Hispanic	7	25.0		
Pacific Islander, Native American or Native Alaskan	1	3.6		
Free or reduced lunch				
Yes	18	64.3		
No	10	35.7		

### 2.2 Selection of Cannabis Product Images

Across the focus groups, participants were shown 15 cannabis edible products available for purchase at retailers in Washington state. A member of the research team, over the age of 21, visited a local cannabis retailer and took photos of more than 25 products after obtaining staff permission. The photographer took photos of sodas, sweet, and savoury edibles that represented multiple product packages. Members of our interdisciplinary research team then selected the final 15 products that would be included in the focus groups, selecting some products that were thought to potentially appeal to youth (e.g., had bright and colourful packaging with images) and some thought to be less appealing (e.g., more muted colours, no visuals). The 15 products were divided into sets of 4–6 photos and each set included at least one sweet edible, one savoury edible and one soda. All final photos included the product package against a black background.

### 2.3 Procedure

A trained moderator facilitated discussion based on the semi‐structured protocol, with a notetaker's assistance. During focus groups, we showed the participants a cannabis edible product image and asked about their initial thoughts, perceptions of appeals used for the product packaging, potential consumers, warning labels and icons, and nutrition facts. Each focus group lasted about 90 minutes.

Our semi‐structured protocol was designed to address: (i) cannabis product appeals; (ii) warning symbols; (iii) serving sizes and nutrition facts; and (iv) comparisons of the products they have seen during the focus group or to other products with which they were familiar (e.g., non‐cannabis snacks or beverages). We opened the discussion with questions about cannabis product appeals, such as, ‘What's the first thing you notice when you see this product?’ The moderator also prompted discussions about the warning symbols shown on the product packages. All products included the ‘Not For Kids’ symbol and Washington Universal Symbol per Washington state requirements. In discussing these warning symbols, most focus groups also talked about other icons or banners included to convey information on the product's effects, cautions and serving size. This led focus groups to discuss what they noticed on product packages in terms of serving size and nutrition facts. Our semi‐structured protocol focused on inquiring whether youth checked or noticed that cannabis edible products have different serving sizes and ingredients (e.g., THC) from non‐cannabis snack products. After reviewing all the products, the moderator asked participants which product they liked the most or the least and why. This allowed the participants to provide final thoughts on cannabis edibles packaging. Additional results related to the appeals perceived in the packaging are provided in a separate manuscript [30].

### 2.4 Analysis

We used Zoom's recording and auto‐transcription features. A member of the research team verified the transcripts prior to coding. We analyzed the data using thematic analysis [31] with MAXQDA. Team members initially familiarized themselves with the data by reviewing the transcripts. We then generated initial codes and identified themes. The team then collaboratively reviewed and refined themes, defined and named themes, and drafted the results.

## 3 Results

### 3.1 Teens May Be Misinterpreting Warning Labels

Participants often misinterpreted the ‘Not For Kids’ symbol. The word ‘kids’ made them connect the symbol to younger children, whom they perceived to be either under 11 or 13 years old, or even younger children, such as toddlers or preschoolers. They did not associate the symbol with themselves because they did not consider themselves ‘kids’. Serena^1^ said, ‘I feel like [the ‘Not for Kids’ symbol] would be very effective if it's like, a younger kid, not like a teenager’. Lily noted: ‘I don't think a lot of kids would refer to themselves as kids, you know, so I don't know if it's, like, if that's super helpful as a deterrent’. This not only shows that participants did not see themselves as kids, but that the word choice could be misleading. In fact, some participants suggested this symbol may not prevent teenagers from using cannabis products but may rather attract the ‘rebels’ who want to feel mature. Josh said, ‘specifically teenagers, who are trying to be older and more mature than kids back in elementary, middle school, and may feel like this isn't for them’.

Participants thought the Poison Control helpline phone number written at the bottom of the symbol was for adults or parents in case they found that a child accidentally consumed cannabis edibles. None of the participants were familiar with the number, and most of them said they would call 911 or their parents if they consumed cannabis edibles by accident. Darlene said, ‘I think it's for adults, like if a kid has eaten one of these it's for adults to like call to figure out what to do’. Some participants said they would not call anyone but wait until the effect disappears. Tyson said, ‘Oh, I'd lay down, like, just try to get through it’. Some thought there was no reason to call because they felt like nothing could be done if a child had already consumed a cannabis edible. For example, Nina said, ‘But I don't know what anyone could do about that, like your kid's already consumed it. I don't know, but maybe that's the point of the number, but I don't, like me personally, I wouldn't do anything’.

Similarly, many participants misinterpreted the Washington Universal Symbol and perceived it as a ‘trendy’ icon rather than a warning label. Although participants seemed to find the Washington Universal Symbol more intuitive and most of them were able to identify the cannabis leaf in the logo, some participants did not recognize it at first. One participant referred to it as an ‘oak leaf’ and realized it was a cannabis leaf later during the discussion. Most of our participants did not perceive the cannabis leaf symbol as a warning sign but as a marketing symbol because they saw local dispensaries using it to ‘attract’ people. Santiago said, I've seen that sign when I'm driving around town. There's a cannabis store there, and I've seen that sign there, like to attract people, ‘this is what we sell it.’ Hence, we found that teens may have misunderstandings about the intentions of the symbol and that some symbols may be perceived as something to look for if teens want to consume cannabis edibles, not necessarily to avoid them.

Participants showed a better understanding of other warning labels or warning statements placed on cannabis edible packages. Most of these labels advised the consumers about the delayed or lasting effects, reminded the consumers not to drive under the influence, and warned about the potential of the product to be habit‐forming. Indeed, participants seemed to understand some easily, like comments about not driving under the influence. However, it was noted that they would not pay much attention to such statements in daily settings. Kayla said, ‘[…] I think the warning labels, like, I would definitely notice them, but I would probably be thinking more about like what kind of ice candy or product it is, and like the flavor’.

### 3.2 Teens Think Warning Labels Are Hidden or Unnoticeable

Although the packages we showed to participants displayed various warning labels, including the ‘Not For Kids’ symbol and the Washington Universal Symbol, most participants did not discuss them until after the moderator called attention to them by magnifying or zooming in on the product packages and asked them to take a closer look. Even though some participants said they noticed the symbols at first glance, they did not stand out enough to be mentioned by the participants in most cases. This was mainly due to their obscurity, such as small label sizes compared to other visual elements on the package (e.g., fonts, flavour‐related images), colours that blend into the background, or the placement of the labels that varied across products, which makes them more difficult to find.

Our participants repeatedly said that both the ‘Not For Kids’ symbol and the Washington Universal Symbol should be a larger size and in contrasting colours that do not blend into the background. Participants also suggested the symbols could be placed at the top of the packages instead of the bottom and the sides of the package. Once they noticed and learned about the warning labels, our participants often pointed out that they are hard to see and even suggested that they are ‘hidden’. Lily said, ‘So [product name] is like a lot bigger than cannabis … It's the first thing you see … The fact that there's like marijuana, that is kind of being hidden’. Some participants also pointed out that the package was so ‘busy’, that the warning labels were not distinctive, and that they would know the product contained cannabis only if they closely inspected what was written on the bottle or the package.

### 3.3 Teens Felt Serving Sizes Were Confusing and Nutrition Labels Were Not a Focus

Nutrition labels on cannabis edibles provide important information for consumers. For Washington, cannabis products' serving size is typically a single unit of a candy or snack product and can be 1/10th of a 12 oz. drink bottle, much smaller than the typical serving size of a non‐infused food item. Therefore, understanding the nutrition labels and serving size information is important for safe consumption. Many of our participants said they do not care about nutrition labels. Austin said: ‘The nutrition facts are on almost everything. So, I probably wouldn't take the time out of my day to look at the nutrition facts’. Nonetheless, participants noticed or identified the serving size easily for some products. They sometimes said that it was hard to identify when the information did not stand out due to other visual aspects of the package, such as colour schemes or the placement of the information. Similar to the warning labels, noticeability was mentioned repeatedly when the participants discussed nutrition labels and serving size information.

Most of the participants were not able to identify the serving size of cannabis edibles or failed to guess how many servings one container might have at the beginning of the discussion. Even when they read the information correctly, they still felt that the serving size was confusing. For example, as they were examining a cannabis‐infused soda product that had ten servings in one bottle, Tyson said, ‘I think this is 10 servings per container … which I think is really weird because … a lot of times drinks like this will … be one per container … it's just really confusing and weird’.

### 3.4 Familiarity May Influence Teens to Review Packaging Less Critically

Teens reported that if the package looked like non‐cannabis products they would likely skip reading the package nutrition information and consume the product like a non‐cannabis product. For example, when we showed a cannabis‐infused soda package that had both warning labels on it, most of the participants did not notice that it contained cannabis. Santiago said, ‘At first sight, it just looks like a regular root beer, so you don't like notice the cannabis symbols or any of those warnings.’ One participant said about a different product: ‘This looks like something your mom buys and maybe, I think a cannabis edible is packaged the same as the ordinary candy’. Also, participants often compared cannabis edibles to non‐cannabis products when discussing serving sizes. For instance, they compared a fruit chew type cannabis edible to a candy popular among youth in the United States, Starbursts, or discussed how they would consume regular pretzels in daily life when they evaluated the pretzel‐type cannabis edible product which indicated one pretzel per serving. Ashley said, ‘It definitely would not be instinctive to just eat one and then be done’.

### 3.5 Personal Interests and Knowledge Impact Teens' Perceptions

When examining the warning labels, some participants had difficulty understanding them due to a lack of knowledge. For example, even though they noticed the cautions about the lasting effects and driving under the influence, they did not fully understand what the lasting effects might mean or see the connection between them to figure out whether one can drive after 2+ hours. Austin said, ‘I also notice at the top, it says, that you have to wait two hours for full effects’. Further, most participants did not discuss that they should not take more than the recommended serving size when they read the warnings about the lasting effects, which suggests they do not understand what to be cautious about. However, participants who already knew driving under the influence is illegal thought the warning label was a good reminder for consumers.

We noted that participants' knowledge of cannabis or cannabis edibles plays a role in examining these packages. Participants who appeared well‐informed about cannabis fully understood what the warnings meant and made correct guesses even if they were not familiar with the warning labels; others were surprised to learn about cannabis as they examined the packages during the discussion. Gabriel saw the warning that the product can be habit‐forming and said, ‘Well, this was like the first time I've seen that's like, it could, you know, be a little addicting and anything you have seen’, and asked his peers if they had ever ‘seen anything like that’.

We also noted that some participants would pay more attention to certain types of information or even actively inspect what is written on the package for personal reasons. For example, Carly was critical about the sugar amount in cannabis edibles and said, ‘I'm an athlete, and like, I really try to eat healthy, so like, when I saw the nutrition facts first … that definitely like doesn't appeal to me’. Some participants also mentioned information that may be relevant to their peers with allergies or dietary restrictions (e.g., gluten‐free), suggesting that they would give more attention to examining the packages if they had specific reasons to do so.

## 4 Discussion

Although the ‘Not for Kids’ symbol and the Washington Universal symbol have requirements about sizing, our participants questioned whether the labels were prominent enough to catch consumers' attention. Attention is an early and important step in the elaboration likelihood model [14], a model that describes message processing, and is needed for people to attend to the presented information. Most participants did not mention the warning labels until after the moderator prompted them to look closely. This is particularly concerning as edible cannabis products may appear similar to non‐cannabis products [32]. Our teen participants also noted similarities to other products in terms of packaging and flavours. Perceived familiarity may lend itself to a lack of central processing around the information presented, as participants may lack motivation to carefully consider product information.

Our research suggests that it is also important to further our understanding of whether packaging symbols are understood as warning labels and noted by intended audiences depending on packaging characteristics. The Washington Universal symbol, which contains an image of a cannabis leaf with the text indicating the product is for people 21 and older, was noted by some participants as similar to advertisements they have seen for cannabis. Images such as the cannabis leaf on hemp products have been suggested to denote a perception that the product is ‘natural’ and therefore appeals to customers [33]. Therefore, it may be useful for regulatory boards to consider ways in which teens' inputs can be brought into the decision‐making process about appeals and perceptions of packaging to help reduce possible unintended effects. Our findings may also be helpful for policymakers to determine how warning labels can be most effective for intended audiences.

The teens we talked with perceived the ‘Not for Kids’ symbol to be primarily included on the packages as a warning for parents of very young children (i.e., those under 13 years old or toddlers), but did not perceive it to be applied to them. This perception is especially noteworthy, as many teenagers may share similar feelings. Adolescence could be a particularly challenging time as many teens seek autonomy from their parents and want to feel grown up, especially if their peers demonstrate increasing autonomy [34]. Therefore, the idea of being ‘kids’ may no longer resonate with this population. Many of our participants did not associate the symbol with being relevant to teens or to themselves. This finding highlights the importance of word choice as it relates to potential audience members; our teens rarely gave attention to this label without prompting, and even with prompting, were likely to discount it as they felt it did not apply to them.

Furthermore, some participants felt this symbol might be attractive to rebellious teens. Future research should consider investigating how teens may misinterpret warning labels, as they may be ineffective or counterproductive. Our participants did, however, mention that the phone number provided on packages as part of the Not For Kids symbol might be relevant if very young children consumed cannabis edibles. Given that some teens participate in caregiving roles as babysitters or may be around young children who could be accidentally exposed to the products, this could be seen as positive. Additional research into which phrasing may most resonate with a variety of people under the age of 21 could be useful.

Our results also indicated that teens would not call the number listed in the ‘Not for Kids’ symbol, which went to Poison Control, even if they or a friend had overconsumed or suffered adverse effects from cannabis edibles; instead, they would rather ‘wait it out’ because they did not perceive there was anything to be done. This, coupled with the fact that teens reported they likely would not pay close attention to serving sizes, highlights that teens may be at an increased risk of negative outcomes from potential misuse. For example, in our study, participants thought one serving of a cannabis‐infused soda would be the entire bottle, even though the bottle included 10 servings. However, consuming one full bottle of a 10‐serving cannabis‐infused soda could lead to a range of adverse effects. Policy makers and advertisers could work to include language on packaging that further highlights the potential benefits of contacting poison control in such situations and focus on product packaging that can further reduce such confusions (e.g., single serving bottles).

Some of our teen participants were unaware of the delay it may take to feel effects from cannabis edibles, which could further lead to misuse and negative effects. The finding that teens also may not call for advice or help after such consumption is further problematic, as it highlights that not only may negative effects occur from overconsumption, but they may not know about resources that could be useful in such situations. Although some participants mentioned they might reach out to a friend or a parent if they were concerned after using a cannabis edible, they may be better served by calling the expert emergency line provided on the package. Therefore, it might be worthwhile to further identify how to make the label relevant to teens in case accidental consumption or overconsumption occurs.

Our results further relate back to the elaboration likelihood model [14], highlighting the role that motivation and ability can play in processing labels on cannabis product packaging. We noted that knowledge and familiarity with products impacted teens' perceptions of the effectiveness of some labels, with those with greater knowledge and familiarity showing a greater understanding of the information being conveyed, likely due to their increased ability to process the content. Additionally, motivation influenced understanding related to nutrition information, with teens who were motivated by health and fitness paying greater attention to some of the features presented in the nutrition labels. Additional research on the role of motivation in the processing of warning labels would be beneficial.

It is important to note the limitations of our work. We chose virtual focus groups as it allowed us to recruit teens from a variety of locations across the state and have them participate in spaces in which they were comfortable. However, it also had the limiting effect of having them view the packaging as images on screens. To mitigate these effects, we showed images of all sides of the products and zoomed in on product aspects. However, it is possible that seeing a bottle in person could also more clearly help participants identify the number of servings in an edible. Given past research that supports single‐serve and unit‐dose packaging as helpful for interpreting serving sizes [35] and our findings among teens that they do not pay attention to serving sizes, it is crucial for policymakers to continue to consider packaging requirements that help potential consumers more easily identify appropriate servings. Some additional limitations to consider include that the study was conducted in Washington state, and products may look different from those in other states where adult‐use cannabis is legal. Lastly, it is important to acknowledge that our study focused primarily on the perceptions of teens but did not directly tie their assessments about warning labels to intentions to use cannabis. Thus, future research should examine how warning labels and nutrition information on cannabis products may be associated with teens' intentions to use cannabis products and cannabis edibles.

## 5 Conclusions

Our qualitative exploration with teens across Washington state provided additional information about how teens interpret cannabis edibles packaging, highlighting the value of adding teen voices to spaces in which policy decisions are happening to further enhance the potential effectiveness of messages designed to caution youth from using cannabis. Teens in our study did not feel that the word ‘kids’ applied to them, highlighting the importance of understanding the perceptions of different groups on the terminology and images used on cannabis edibles packaging, as teens may be exposed to such products. Similarly, some teens perceived the cannabis leaf included in the Washington Universal Symbol as an advertisement for cannabis products. Additional research could be helpful in furthering our understanding of what content may provide knowledge that products are cannabis‐infused without appearing as advertisements to young people, especially given that knowledge and product familiarity may impact how teens perceive cannabis product packaging. Finally, educational efforts that further increase packaging literacy would be beneficial for youth.

## Author Contributions

**Jessica Fitts Willoughby:** conceptualisation; data collection; data analysis; manuscript preparation; interpretation of data. **Stacey J. T. Hust:** conceptualisation; data collection; data analysis; manuscript preparation; interpretation of data. **Soojung Kang:** data collection; data analysis; manuscript preparation. **Leticia Couto:** data collection; data analysis; manuscript preparation. **Ron Price:** data collection; data analysis; manuscript preparation. **Christina Griselda Nickerson:** data collection; data analysis; manuscript preparation. **Opeyemi Johnson:** data collection; data analysis; manuscript preparation. **Sarah Ross‐Viles:** conceptualisation; data collection; critical revision of manuscript.

## Conflicts of Interest

The authors declare no conflicts of interest.

## Data Availability

The data that support the findings of this study are available on request from the corresponding author. The data are not publicly available due to privacy or ethical restrictions.

## Endnotes

^1^
All names presented are pseudonyms.
